# Comparative Study Between Extended-View Totally Extraperitoneal Rives–Stoppa Repair and Intraperitoneal Onlay Mesh Plus Repair for Ventral Abdominal Wall Hernias: A Randomized Controlled Trial

**DOI:** 10.7759/cureus.92779

**Published:** 2025-09-20

**Authors:** Kavita Sijwali, Sanjay Kala, Ramendra K Jauhari

**Affiliations:** 1 General Surgery, Ganesh Shankar Vidyarthi Memorial Medical College, Kanpur, IND

**Keywords:** anterior abdominal wall reconstruction, e-tep rs, intraperitoneal onlay mesh repair (ipom) plus, minimally invasive surgery, ventral and incisional hernia

## Abstract

Introduction

Ventral hernias are among the most common abdominal wall defects encountered in surgical practice and are increasingly managed laparoscopically due to lower postoperative morbidity and faster recovery times. While the intraperitoneal onlay mesh plus (IPOM Plus) technique is widely practiced, the extended totally extraperitoneal Rives-Stoppa (e-TEP RS) approach enables extraperitoneal mesh placement and may offer improved outcomes. This study aims to compare the two techniques regarding operative parameters, complications, and patient-reported outcomes.

Methods

A prospective randomized controlled trial was conducted at Ganesh Shanker Vidyarthi Mandir (GSVM) Medical College, Kanpur, from February 2024 to April 2025. A total of 174 patients with primary or incisional ventral hernias were randomized into two groups: e-TEP RS (n = 87) and IPOM Plus (n = 87). Outcomes assessed included operative time, pain using Visual Analogue Scale (VAS) scores, hospital stay, return to activity, complications (seroma, hematoma, surgical site infections, and recurrence), chronic pain, quality of life, and conversion rate over six months. This study was registered on ClinicalTrial.gov with the identifier NCT07055464.

Results

The e-TEP RS group had a significantly longer operative time (170.2 ± 14.0 min vs. 90.7 ± 9.9 min; *p* < 0.001) but demonstrated lower postoperative and chronic pain scores (*p* < 0.001), shorter hospital stay (1.8 ± 0.6 days (n = 87) vs. 3.2 ± 1.1 days; *p* < 0.001), and earlier return to activity (4.8 ± 1.2 days vs. 7.5 ± 2.0 days; *p* < 0.001). Quality of life at three and six months was significantly better (*p* < 0.001).Peritoneal breach occurred only in e-TEP RS (nine cases, 10.3%), while bleeding > 50 mL was more frequent in IPOM Plus (18 cases, 20.7% vs. six cases, 6.9%; *p* = 0.014). Recurrence, surgical site infection, and seroma rates were lower in e-TEP RS but not statistically significant. Conversion to IPOM Plus was needed in two e-TEP RS cases (2.3%) due to intraoperative challenges.

Conclusion

Despite longer operative time, the e-TEP RS technique demonstrated superior postoperative outcomes and may be preferred in appropriate surgical candidates. The IPOM Plus technique remains a viable alternative in cases where extraperitoneal dissection is not feasible.

## Introduction

Ventral hernia is characterized by the protrusion of abdominal contents, including bowel loops, omentum, or preperitoneal fat, through a defect or weakened area of the abdominal wall. These hernias may arise due to congenital anomalies, acquired defects in the musculature, or as a complication following abdominal surgery. The European Hernia Society broadly classifies ventral hernias into primary (congenital or spontaneous) and incisional types [1]. Primary ventral hernias include midline hernias (e.g., epigastric, umbilical) and lateral hernias (e.g., lumbar, spigelian), while incisional hernias occur at sites of previous abdominal incisions and are further subclassified based on location [2].

Ventral hernia repair is one of the most frequently performed operations in general surgery. The key goals are to restore abdominal wall integrity, prevent recurrence, and reduce the risk of complications such as obstruction or strangulation. Surgical techniques include primary suture repair and prosthetic reinforcement with mesh, with the latter significantly lowering recurrence rates. Mesh placement strategies include onlay, inlay, sublay, preperitoneal, and intraperitoneal positions, each associated with distinct advantages and limitations [3].

Among open techniques, the Rives-Stoppa repair is considered the gold standard. This method involves retrorectus placement of mesh to restore the linea alba and reinforce the abdominal wall, offering a durable repair with a reduced risk of infection and adhesion formation [4].

In 1993, LeBlanc and Booth pioneered the laparoscopic intraperitoneal onlay mesh (IPOM) technique, which enabled minimally invasive hernia repair using an intraperitoneally placed mesh [5]. While IPOM offered benefits like faster recovery and reduced wound complications, it also introduced concerns such as prosthetic erosion, bowel adhesions, and chronic pain due to tacks or transfascial sutures [6].

To address the drawbacks of conventional laparoscopic hernia repairs, particularly the risk of visceral adhesions and suboptimal mesh positioning, several advanced techniques have been developed to allow mesh placement in anatomically favorable planes while minimizing visceral contact. These include transabdominal preperitoneal repair (TAPP), where the mesh is placed in the preperitoneal space via the peritoneal cavity [7], and totally endoscopic sublay (TES), which involves endoscopic placement of mesh in the retrorectus space. The endoscopic mini/less open sublay (EMILOS) technique combines laparoscopic and open methods to achieve sublay mesh positioning [8]. Similarly, the retro-rectus sublay repair places the mesh behind the rectus muscles but anterior to the posterior rectus sheath. Another method, the subcutaneous onlay laparoscopic approach (SCOLA), utilizes laparoscopic access for subcutaneous mesh placement [9]. Among these, the extended-view totally extraperitoneal (e-TEP) repair stands out for offering a wide extraperitoneal working space, facilitating optimal mesh deployment without breaching the peritoneal cavity.

A further innovation is the e-TEP RS (extended-view totally extraperitoneal Rives-Stoppa) technique, which incorporates minimally invasive access with the robust anatomical principles of the Rives-Stoppa method. This technique enables retromuscular mesh placement while avoiding mesh contact with intra-abdominal organs, thus minimizing risks of adhesion, erosion, and infection [10,11].

Recent comparative studies between IPOM plus and e-TEP RS repairs have shown that the latter results in significantly lower postoperative pain, reduced mesh-related complications, and quicker return to daily activities. The recurrence rates are also favorably low with e-TEP RS [12].

Our study aims to contribute to this growing body of evidence by comparing IPOM plus and e-TEP RS techniques for uncomplicated ventral hernias. Through analysis of return to normal activites, perioperative outcomes, recurrence, and complication rates, we aim to support evidence-based decision-making for optimal surgical management [13,14].

## Materials and methods

Study design and registration

This prospective, randomized controlled study was conducted in the Department of General Surgery, Ganesh Shankar Vidyarthi Memorial (GSVM) Medical College, Kanpur, between February 2024 and April 2025. The study included 174 patients aged over 18 years, of either sex, diagnosed with primary or incisional ventral hernia and deemed suitable for elective laparoscopic repair. Only patients who provided written informed consent were enrolled. Participation was entirely voluntary, and patients were free to withdraw at any point without any compromise to their clinical care. Institutional ethical clearance was obtained from the Ethics Committee for Biomedical Health and Research of GSVM Medical College, Kanpur, prior to study initiation under Protocol No. EC/BMHR/2024/37. This study was registered on ClinicalTrial.gov with the identifier NCT07055464. No changes were made to the trial methods after the commencement of the study.

Study sample

The sample size was calculated based on the primary outcome of the study, which was the time to return to normal activities following hernia repair. Assuming a mean difference of two days between the two groups, with a standard deviation of approximately 6.0 days, a power of 80%, and a two-tailed alpha level of 0.05, the minimum required sample size was determined using the formula for comparing two independent means. This yielded a required sample of 78 patients per group. To account for a potential 10% dropout rate or loss to follow-up, the final sample size was adjusted to 87 patients in each group, resulting in a total of 174 patients for the study. A total of 174 patients diagnosed with ventral hernia were included in the study. Randomization was performed using an even-odd allocation method Group I (odd-numbered patients) underwent e-TEP RS hernia repair. Group II (even-numbered patients) underwent IPOM Plus hernia repair. No interim analysis was conducted and no predefined stopping rules were applied. 

Inclusion and exclusion criteria

Patients aged between 18 and 60 years presenting with uncomplicated primary or incisional ventral hernias, having a defect size between 2.0 cm and 5.0 cm, and scheduled for elective (non-emergency) laparoscopic repair were included in the study. Exclusion criteria comprised patients with defect size >5 cm, lateral hernias, or complicated hernias (irreducible, obstructed, or strangulated). Patients with a history of prior hernia surgery or recurrence, those unfit for general anesthesia, on anticoagulant therapy, in poor general condition, pregnant or lactating women, and individuals who did not provide informed consent were excluded from the trial.

Study outcomes

The primary outcome of this randomized controlled trial was the time to return to normal activities, defined as the duration (in days) taken by the patient to resume routine daily or occupational tasks without discomfort. This parameter was selected to reflect postoperative functional recovery and overall rehabilitation.

Secondary outcomes included assessment of postoperative and chronic pain using the Visual Analog Scale (VAS), recorded at 24 hours, seven days, three months, and six months postoperatively. Chronic pain was defined as pain persisting beyond three months after surgery. Additional secondary measures included operative time (from skin incision to closure, in minutes), hospital stay duration (in days), and intraoperative complications such as peritoneal tear and intraoperative bleeding exceeding 50 mL. Postoperative complications evaluated were surgical site infection (SSI), defined according to Centers for Disease Control and Prevention (CDC) guidelines as infections occurring at the surgical site within 30 days of the procedure, along with seroma and hematoma, confirmed either clinically or by ultrasound. Recurrence was defined as clinical or radiological evidence of hernia at the operated site during the six-month follow-up period. Feasibility of each surgical technique was also evaluated, based on successful completion without the need for conversion. Finally, quality of life was assessed at three and six months postoperatively using the World Health Organization Quality of Life-BREF (WHOQOL-BREF) questionnaire. There were no changes to the trial outcomes after the trial commenced.

Statistical analysis

All data were entered into a Microsoft Excel spreadsheet (Microsoft Corp., USA), and the final analysis was conducted using IBM SPSS Statistics for Windows, version 25.0 (released 2017, IBM Corp., Armonk, NY). Mean and standard deviation were used to describe continuous variables with a normal distribution, while median and interquartile range (IQR) were used for continuous variables with a non-normal distribution. Categorical variables were summarized as counts and percentages. For comparisons between two independent groups, the Student’s t-test was used for normally distributed continuous variables, and the Mann-Whitney U test was used for non-normally distributed continuous variables. Categorical variables were compared using Pearson’s Chi-square test or Fisher’s exact test, as appropriate. A p-value of <0.05 was considered statistically significant. No subgroup or adjusted analyses were planned or performed, as baseline demographic and clinical variables were comparable between the two groups.

## Results

A total of 174 patients were included, with 87 patients each in the e-TEP RS and IPOM Plus groups. Both groups were comparable in demographic and baseline parameters. The mean age was similar between the e-TEP RS and IPOM Plus groups (45.6 ± 8.2 years (n = 87) vs. 46.8 ± 7.9 years (n = 87); p = 0.52), with a male predominance observed in both (58 cases, 66.7% vs. 55 cases, 63.3%).Comorbidities such as hypertension (26 cases, 30.0% vs. 29 cases, 33.3%), diabetes (23 cases, 26.7% vs. 20 cases, 23.3%), and obesity (17 cases, 20.0% vs. 14 cases, 16.7%) showed no significant differences (all p > 0.05). Mean BMI values were also comparable (p = 0.68), reflecting balanced baseline health status. Regarding hernia characteristics, primary hernias were slightly more common in the IPOM Plus group (48 cases, 55.2% vs. 52 cases, 59.8%), while incisional hernias were more frequent in the e-TEP RS group (39 cases, 44.8% vs. 35 cases, 40.2%), but these differences were not statistically significant (p = 0.74). The mean hernia defect size was also similar between the groups (3.8 ± 0.9 cm (n = 87) vs. 3.5 ± 0.8 cm (n = 87); p = 0.49). These findings confirm that the two groups were demographically and clinically comparable at baseline, allowing for unbiased outcome assessment (Table 1).

**Table 1:** Baseline demographic and clinical characteristics of patients in the e-TEP RS and IPOM Plus groups NS = not significant (p > 0.05); e-TEP RS: extended totally extraperitoneal Rives-Stoppa, IPOM Plus: intraperitoneal onlay mesh plus

Variable	e-TEP RS (n = 87)	IPOM Plus (n = 87)	p-value
Mean age (years)	45.6 ± 8.2	46.8 ± 7.9	0.52
Male, n (%)	58 (66.7)	55 (63.3)	NS
Female, n (%)	29 (33.3)	32 (36.7)	NS
Hypertension (HTN), n (%)	26 (30.0)	29 (33.3)	0.76
Diabetes mellitus (DM), n (%)	23 (26.7)	20 (23.3)	0.81
Obesity (BMI > 30 kg/m²), n (%)	17 (20.0)	14 (16.7)	0.67
Primary hernia, n (%)	52 (60.0)	48 (55.0)	0.74
Incisional hernia, n (%)	35 (40.0)	39 (45.0)	0.74
Mean hernia size (cm)	3.5 ± 0.8	3.8 ± 0.9	0.49

Postoperative pain and chronic pain

Postoperative pain was evaluated at different time points using the VAS scale (Table 2). Patients undergoing e-TEP RS reported significantly lower pain scores at all follow-ups (p < 0.001). Chronic pain was assessed using the VAS at three and six months postoperatively (Table 2). At three months, mean VAS scores were significantly lower in the e-TEP RS group (1.5 ± 0.8) compared to the IPOM Plus group (2.7 ± 1.0; p < 0.001). This difference remained significant at six months (0.8 ± 0.5 vs. 1.6 ± 0.6; p < 0.001).

**Table 2:** Comparison of operative, postoperative, and quality of life outcomes between the e-TEP RS and IPOM Plus techniques (n = 87 each) e-TEP RS: extended totally extraperitoneal Rives-Stoppa, IPOM Plus: intraperitoneal onlay mesh plus, VAS: Visual Analogue Scale (0–10 scale for pain), QOL: quality of life, WHOQOL-BREF: World Health Organization Quality of Life-BREF Questionnaire, SD: standard deviation, SSI: surgical site infection, post-op: postoperative

Parameter	e-TEP RS (n = 87)	IPOM Plus (n = 87)	p-value
Operative time (min, Mean ± SD)	170.2 ± 14.0	90.7 ± 9.9	<0.0001
Hospital stay (days, Mean ± SD)	1.8 ± 0.6	3.2 ± 1.1	<0.001
Return to normal activity (days)	4.8 ± 1.2	7.5 ± 2.0	<0.001
Post-op pain (VAS at 24 hours)	4.5 ± 1.2	6.3 ± 1.4	<0.001
Post-op pain (VAS at seven days)	2.8 ± 1.0	4.1 ± 1.3	0.004
Chronic pain (VAS at three months)	1.5 ± 0.8	2.7 ± 1.0	<0.001
Chronic pain (VAS at six months)	0.8 ± 0.5	1.6 ± 0.6	<0.001
Bleeding >50 mL (%)	6(6.9%)	18 (20.7%)	0.014
Peritoneal tear (%)	9 (10.3%)	0	0.002
Surgical site infection (%)	2 (2.3%)	7 (8%)	0.168
Seroma formation (%)	6 (6.9%)	13 (14.9%)	0.127
Hematoma formation (%)	0	3 (3.4%)	0.246
Recurrence rate (%)	6 (6.9%)	9 (10.3%)	0.427
Conversion to other technique (%)	2 (2.3%)(to IPOM Plus)	0	0.45
QOL score (WHOQOL-BREF) three months	82.5 ± 5.2	76.3 ± 6.1	<0.0001
QOL score (WHOQOL-BREF) six months	89.2 ± 4.8	80.6 ± 5.9	<0.0001

Operative time and intraoperative complications

The mean operative time was significantly longer in the e-TEP RS group (170.2 ± 14.0 minutes) compared to the IPOM Plus group (90.7 ± 9.9 minutes; p < 0.0001), likely due to the increased technical demands of creating the retrorectus space and closing the posterior sheath. Intraoperative complications varied between the groups. Peritoneal tears occurred in nine (10.3%) of e-TEP RS cases but were not observed in the IPOM Plus group. Conversely, bleeding exceeding 50 mL was significantly more frequent in the IPOM Plus group (18 vs. 6; 20.7% vs. 6.9%) with significant p value (p = 0.014), potentially related to more extensive intraperitoneal dissection and mesh fixation.

Postoperative complications and recovery

The surgical site infection (SSI) rate was lower in the e-TEP RS group (2; 2.3%) compared to the IPOM Plus group (7; 8.0%), although this difference was not statistically significant (p = 0.168), likely due to the extraperitoneal mesh placement reducing contamination risk. Seroma formation occurred more frequently in the IPOM Plus group (13; 14.9%) than in the e-TEP RS group (6; 6.9%; p = 0.127), while hematoma was reported only in the IPOM Plus group (3; 3.4%). The mean hospital stay was significantly shorter in the e-TEP RS group (1.8 ± 0.6 days) than in the IPOM Plus group (3.2 ± 1.1 days; p < 0.001). In addition, patients in the e-TEP RS group returned to normal activities earlier (4.8 ± 1.2 vs. 7.5 ± 2.0 days; p < 0.001).

**Figure 1:**
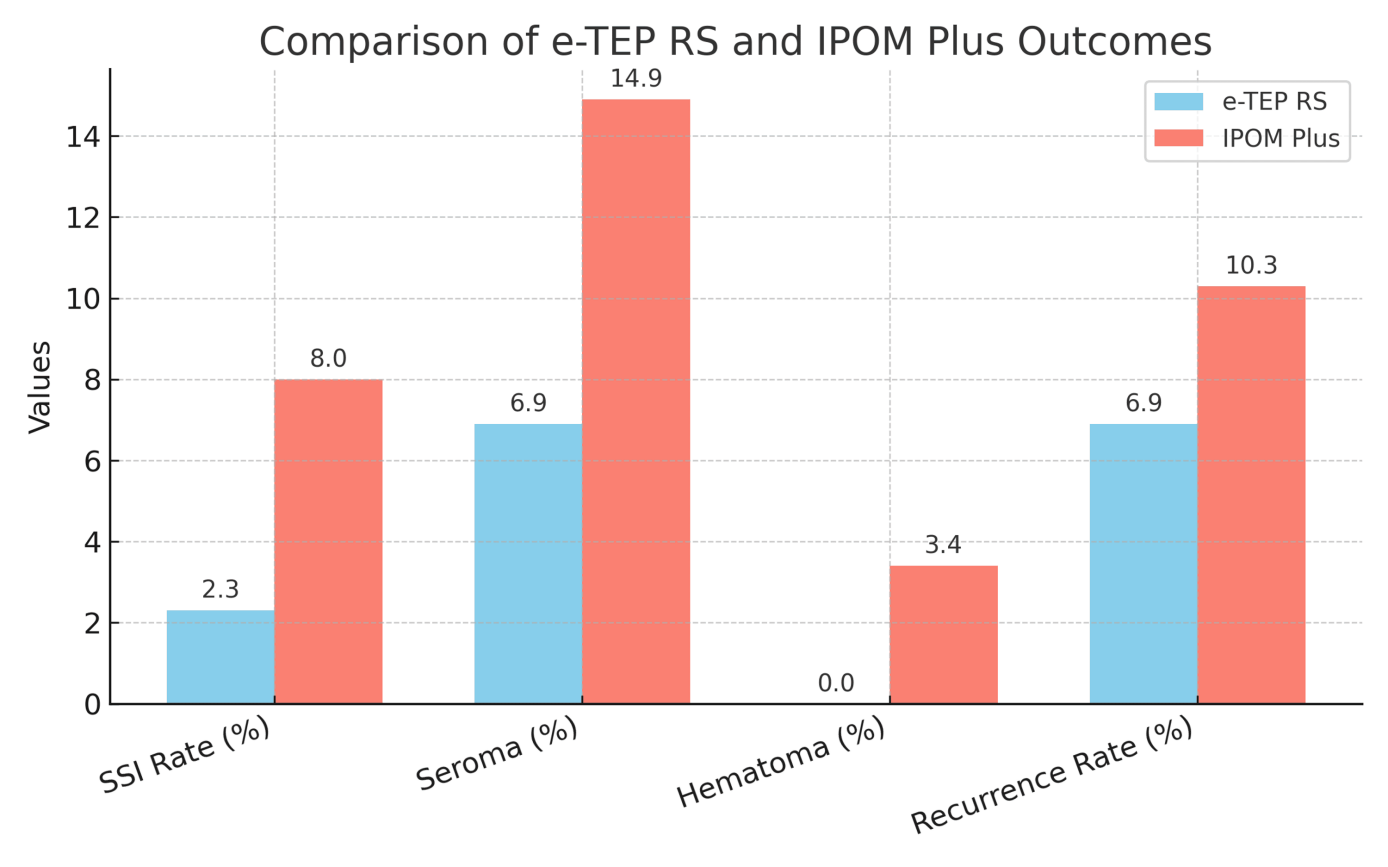
Comparison of postoperative outcomes between the e-TEP RS and IPOM Plus techniques This bar chart compares key postoperative outcomes between the two surgical techniques. Parameters include the following: 1) surgical site infection (SSI) rate (%): incidence of postoperative wound infections; 2) seroma (%): incidence of fluid accumulation at the surgical site, 3) hematoma (%): incidence of blood collection at the surgical site; 4) return to activities (days): time taken by patients to resume normal daily activities; 5) recurrence rate (%): percentage of patients experiencing hernia recurrence. Blue bars: e-TEP RS group; red bars: IPOM Plus group e-TEP RS: extended totally extraperitoneal Rives-Stoppa, IPOM Plus: intraperitoneal onlay mesh plus

**Figure 2:**
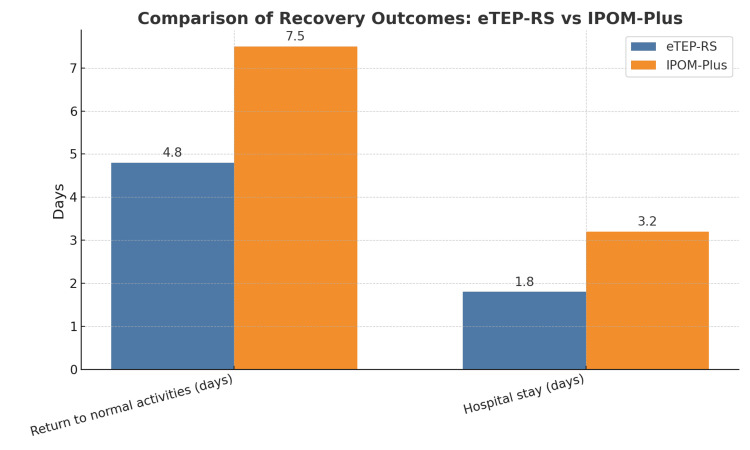
Comparison of recovery outcomes between the e-TEP RS and IPOM Plus techniques This bar chart compares key postoperative outcomes between the two surgical techniques. Parameters include the following: 1) Return to normal activities (days): time taken by patients to resume normal daily activities; 2) hospital stay (days): duration of postoperative hospital admission. Blue bars: e-TEP RS group; orange bars: IPOM Plus group e-TEP RS: extended totally extraperitoneal Rives-Stoppa, IPOM Plus: intraperitoneal onlay mesh plus

Recurrence rates were lower in the e-TEP RS group (6; 6.9%) compared to the IPOM Plus group (9; 10.3%), although this difference was not statistically significant (p = 0.427), possibly due to the more secure retrorectus mesh placement. Quality of life, assessed using the WHOQOL-BREF questionnaire, showed significantly better physical health and pain scores in the e-TEP RS group (82.5 ± 5.2) compared to the IPOM Plus group (76.3 ± 6.1; p < 0.0001) at three months. At six months, the overall functional outcome and patient satisfaction scores were also significantly higher in the e-TEP RS group (89.2 ± 4.8) compared to the IPOM Plus group (80.6 ± 5.9; p < 0.0001) (Figures 1, 2).

Feasibility

The e-TEP RS technique was feasible in 97.7% of patients. In two cases (2.3%), conversion to IPOM Plus was required due to challenging dissection in the retrorectus space. Notably, no conversions to open surgery occurred in either group, supporting the safety and practicality of both minimally invasive approaches.

Overall comparison of key outcomes

To summarize the major findings, Table 2 presents an overall statistical comparison of key postoperative outcomes between e-TEP RS and IPOM Plus. These findings confirm that e-TEP RS provides significant advantages over IPOM Plus in terms of postoperative pain reduction, shorter hospital stays, and faster recovery. No ancillary analyses were conducted in this study.

## Discussion

This comparative study between the e-TEP RS and IPOM Plus techniques for ventral hernia repair demonstrates that both are effective and safe; however, e-TEP RS offers clear advantages in postoperative pain reduction, faster recovery, and improved quality of life. These results support the growing body of literature favoring extraperitoneal mesh placement.

Operative time and technical aspects

The e-TEP RS technique had a significantly longer operative time, primarily due to its technical complexity. Meticulous dissection of the retrorectus space, contralateral crossover, posterior sheath closure, and mesh placement all contribute to the extended duration. Our findings align with those of Belyansky et al. (2018) [15] and Li et al. (2022) [16], who also reported improved postoperative recovery in e-TEP RS repair. However, operative time remains a limitation due to the learning. However, with increasing experience, the operative time is expected to decrease. By contrast, IPOM Plus involves fewer dissection planes and allows quicker mesh deployment but at the cost of higher postoperative complications like pain and seroma formation.

Postoperative pain and recovery

A major benefit of e-TEP RS is the significantly lower postoperative pain at all measured time points. Unlike IPOM Plus, which typically involves transabdominal fixation, e-TEP RS avoids intraperitoneal contact and often requires no tack fixation. This reduces nerve irritation and inflammation. Lehr et al. (2001) [17] and Harsløf et al. (2021) [18] have documented similar findings, associating tacking with chronic pain syndromes. 

Complications: seroma, hematoma, and infection

Although not statistically significant, seroma was more frequent in the IPOM Plus group. This aligns with Taşdelen et al.'s findings (2021) [19], who reported lower seroma rates in e-TEP RS due to better closure of potential dead space. Hematoma and surgical site infection rates were low and comparable between groups, suggesting both techniques are safe when performed with appropriate technique.

Hospital stay and return to function

Patients undergoing e-TEP RS had a significantly shorter hospital stay and returned to normal activities approximately three days earlier than the IPOM Plus group. These outcomes are supported by Slavu et al. (2024) [20] and Bittner et al. (2019) [21], who found enhanced recovery profiles for extraperitoneal approaches. Faster recovery translates to improved patient satisfaction and reduced healthcare utilization.

Recurrence and short-term outcomes

While recurrence rates were slightly lower in the e-TEP RS group (six cases, 6.9% vs. nine cases, 10.3%), the difference was not statistically significant. Both e-TEP RS and IPOM Plus procedures included fascial defect closure, and given the six-month follow-up period, no conclusions regarding long-term durability can be made.

Chronic pain and quality of life

Chronic pain was lower in the e-TEP RS group and WHO-QOL BREF scores at three and six months were significantly better (p < 0.001). These findings support the advantages of retromuscular mesh placement in terms of reducing chronic pain and improving short-term quality of life.

Clinical implications

This study reinforces the growing evidence supporting e-TEP RS as a preferable technique for ventral hernia repair in appropriately selected patients. It offers distinct advantages such as reduced postoperative pain, shorter hospital stay, faster return to normal activities, and improved long-term quality of life. However, the technique requires a steeper learning curve and longer operative time, emphasizing the importance of structured training and careful patient selection. In patients with multiple prior abdominal surgeries, dense adhesions, or contraindications to extraperitoneal dissection, IPOM Plus remains a valid and safe alternative. Ongoing efforts to optimize mesh placement and reduce fixation-related discomfort may help mitigate the risk of chronic pain associated with IPOM Plus.

Study limitations

This study has several limitations. First, it was conducted at a single tertiary care center, which may limit the generalizability of the findings to other populations or healthcare settings. Second, the sample size, though adequate for primary outcomes, may not be large enough to detect statistically significant differences in less common complications such as hematoma or recurrence. Third, the follow-up duration of six months may not capture late recurrences or long-term complications, which are essential in hernia repair outcomes. In addition, while efforts were made to minimize observer bias, the lack of blinding could have influenced subjective assessments such as return to normal activities and quality of life scores. Further multicenter studies with larger cohorts and longer follow-up are warranted to validate these results.

## Conclusions

This study highlights the clinical advantages of the e-TEP RS technique over IPOM Plus in ventral hernia repair. While both techniques are safe and effective, e-TEP RS demonstrated superior clinical outcomes in terms of pain reduction, faster recovery, and improved quality of life, making it a promising alternative for appropriately selected patients. Although the operative time was longer with e-TEP RS due to its technical complexity, this is expected to improve with surgical experience. Recurrence rates and seroma formation were comparable between the two groups. However, e-TEP RS was associated with a lower incidence of chronic pain and provided better anatomical restoration, offering stronger long-term support. The need for conversion to IPOM Plus was minimal, underscoring the importance of training in extraperitoneal approaches. Overall, e-TEP RS appears to be a durable, patient-centered option for uncomplicated ventral hernias and may represent the future standard for minimally invasive hernia repair.
